# Prostate Stem Cell Antigen Expression in Radical Prostatectomy Specimens Predicts Early Biochemical Recurrence in Patients with High Risk Prostate Cancer Receiving Neoadjuvant Hormonal Therapy

**DOI:** 10.1371/journal.pone.0151646

**Published:** 2016-03-16

**Authors:** Sung Han Kim, Weon Seo Park, Sun Ho Kim, Boram Park, Jungnam Joo, Geon Kook Lee, Jae Young Joung, Ho Kyung Seo, Jinsoo Chung, Kang Hyun Lee

**Affiliations:** 1 Department of Urology, Center for Prostate Cancer, Research Institute and Hospital, National Cancer Center, Goyang, Korea; 2 Department of Pathology, Center for Prostate Cancer, Research Institute and Hospital, National Cancer Center, Goyang, Korea; 3 Department of Radiology, Center for Prostate Cancer, Research Institute and Hospital, National Cancer Center, Goyang, Korea; 4 Biometric Research Branch, Research Institute and Hospital, National Cancer Center, Goyang, Korea; University Hospital Carl Gustav Carus Dresden, GERMANY

## Abstract

We aimed to identify tissue biomarkers that predict early biochemical recurrence (BCR) in patients with high-risk prostate cancer (PC), toward the goal of increasing the benefits of neoadjuvant hormonal therapy (NHT). In 2005–2012, prostatectomy specimens were collected from 134 PC patients who had received NHT and radical prostatectomy. The expression of 13 tissue biomarkers was assessed in the specimens via immunohistochemistry. Time to BCR and factors predictive of BCR were determined by using the Cox proportional hazards model. During the follow-up period (median, 57.5 months), 67 (50.0%) patients experienced BCR. Four (3.0%) patients were tumor-free in the final pathology assessment, and 101 (75.4%) had negative resection margins. Prostate stem cell antigen (PSCA) was the only significant prognostic tissue biomarker of BCR [hazard ratio (HR), 2.58; 95% confidence interval (CI), 1.06–6.27; p = 0.037] in a multivariable analysis adjusted by the clinicopathological variables that also significantly predicted BCR; these were seminal vesicle invasion (HR, 2.39; 95% CI, 1.32–4.34), initial prostate serum antigen level (HR 1.01; 95% CI, 1.001–1.020), prostate size (HR, 0.93; 95% CI, 0.90–0.97), and the Gleason score of preoperative biopsies (HR, 1.34; 95% CI, 1.01–1.79). We suggest that PSCA is a useful tissue marker for predicting BCR in patients with high risk PC receiving NHT and radical prostatectomy.

## Introduction

Owing to advances in diagnostic screening and therapeutic procedures including surgery, clinical outcomes in prostate cancer (PC) have recently improved. However, non-metastatic advanced PC that extends beyond the prostate gland (defined as clinical T3 and 4 stages) has a relatively high chance of biochemical recurrence (BCR) after radical prostatectomy (RP) and a 5-year BCR-free survival rate of only 10–40% [1, 2].

To improve the clinical outcome, as assessed via changes in local disease burden, of locally advanced PCs and high risk PCs, neoadjuvant hormonal therapy (NHT) has been performed before RP in the past two decades [3]. Doing so has improved the pathological outcome of undetected micro-metastases in clinical trials with different endpoints owing to a more complete resection with a higher possibility of organ-confined disease, a lower possibility of extracapsular extension, positive surgical margins, and lymph node involvement, and a greater reduction in testosterone and prostate-specific antigen (PSA) levels [4]. However, NHT before RP does not improve overall survival or progression-free survival according to a recent systematic review and meta-analysis [5].

Pathological examination of RP specimens after NHT shows that NHT clearly effects the morphology and immunohistochemical staining patterns of prostate adenocarcinomas. Morphological signs of tumor regression such as apoptosis-induced atrophy of non-neoplastic and neoplastic prostatic epithelium are consistently seen; however, they complicate the recognition and grading of treated carcinomas, which is an objective means of assessing the success of therapy in histopathological follow-ups monitoring local PC regression.

Toward the goal of identifying significant prognostic factors for BCR, we examined a set of tissue biomarkers in whole-mounted RP specimens, as well as clinicopathological variables, in patients with high risk PC who received NHT.

## Materials and Methods

### Ethical statements

All study protocols were conducted according to the ethical guidelines of the World Medical Association Declaration of Helsinki Ethical Principles for Medical Research Involving Human Subjects. This study was approved by the Institutional Review Board of the Research Institute and Hospital National Cancer Center (IRB No. NCCNCS 05–049). All enrolled patients provided written informed consent.

### Patient selection

From February 2005 to December 2012, the samples of 168 patients with high risk PC who underwent RP with NHT at the Center for Prostate Cancer at the National Cancer Center in Goyang, Korea were prospectively collected. Patients who were lost to follow-up, followed-up for less than 1 year, had missing information, or had received salvage prostatectomy, chemotherapy, or invasive prostate therapy were excluded before collection. After collection, 34 patients were excluded because their adjuvant therapeutic history included hormonal therapy (N = 23) or radiotherapy (RT) (N = 11) before BCR, resulting in a final total of 134 patients. Based on the criteria of D’Amico et al. [2], high risk PC was defined as clinical stage ≥T2c, a PSA level >20 ng/mL, or a Gleason score ≥8 in the present study.

### NHT regimen and diagnostic and follow-up protocols

The NHT regimen consisted of a three- to six-month therapy of luteinizing hormone-releasing hormone agonist (LHRH; 7.5 mg leuprolide acetate per month or 10.8 mg goserelin acetate every 3 months), with/without a nonsteroidal anti-androgen (e.g., 50 mg bicalutamide per day). RP was performed with standard pelvic lymph node dissection. No patients in the study cohort (N = 134) had received preoperative therapy with novel endocrine agents such as enzalutamide or abiraterone.

Serum PSA levels were measured in all patients at initial diagnosis, during NHT, and at follow-ups according to the follow-up protocol after RP [6]. Clinical T stages were determined at initial diagnosis via digital rectal examination and imaging. Enlarged lymph nodes and distant metastases were detected via prostate magnetic resonance imaging with/without abdominal computed tomography, and the scans were interpreted by a uroradiologist with 15 years of experience (Sun Ho Kim). Tumor grade and pathologic T stage (ypT) were determined by an uro-oncology pathologist with 20 years of experience (WSP). Preoperative biopsied tumors were graded according to the Gleason scoring system, and staging was performed according to the 2009 TNM classification and the guidelines of the 2005 International Society of Urological Pathology consensus conference and of the WHO classification[7, 8]. Staging is not used for post-hormone therapy specimens according to a worldwide consensus. BCR was considered synonymous with disease recurrence and was defined as a serum PSA level ≥0.2 ng/mL in two consecutive evaluations.

When nadir PSA decreases to undetectable level after surgery, no additional treatment added. When the PSA nadir did not decrease below 0.2 ng/ml following RP, adjuvant ADT or RT was added at the physician’s discretion. In all patients, serum PSA levels were measured every 3 months in the first year after surgery, semiannually in the second to fifth year, and annually thereafter. When BCR was observed, radiologic imaging (magnetic resonance imaging, computed tomography, or a bone scan) was performed if needed. Salvage RT or androgen-deprivation therapy was recommended for patients who experienced local recurrence or distant metastasis.

### Immunohistochemistry and interpretation

Seven tissue microarray (TMA) blocks were prepared as previously described for histology and immunohistochemistry (IHC) of thirteen markers [9]: Ki-67, c-Erb-2, PTEN, ERG, cyclin D1, vascular endothelial growth factor, epidermal growth factor receptor, Rb, prostate stem cell antigen (PSCA), p53, Bcl-2, cyclooxygenase-2, and PMS2. After deparaffinization, heat-induced antigen retrieval of the tissue biomarkers (see Table 1 for specific details), sectioning, and staining of the blocks, localization and distribution were assessed. Residual carcinoma cells on slides were carefully examined, with reference to previously published reports [10–12]. Suitable areas for tissue retrieval were marked on standard hematoxylin and eosin (HE)-stained sections, punched out of the paraffin blocks, and inserted into recipient blocks [13]. After HE staining, all tissues were reviewed to confirm the diagnosis. Sufficient amounts of tumor and normal tissue were used to ensure consistency in the morphological assessments.

**Table 1:** Primary antibodies used in this study.

Antibody	Clone name	Ab dilution-incubation time—BMXT condition*	Vendor
Cerb-2	4B5	R.T.U^+^, 20min, Mild	VENTANA
Cyclin D1	p2D11F11	x40, 44min,STD	Novocastra
VEGF	G153-694	x500, 32min,Mild	Pharmingen
EGFR	31G7	x200, 32min,Protease 4min	Invitrogen
Rb loss	G3-245	x400, 32min,Mild	Pharmingen
PSCA	poly	x500, 32min, Mild	Zymed
p53	Bp53-11	R.T.U, 32min,Mild	VENTANA
Bcl-2	124	R.T.U, 32min,STD	DAKO
Cox-2	H-62	x300, 32min,Mild	Cayman
PMS2	A16-4	x80, 2hr,STD	Pharmingen
Ki-67	MIB-1	x200, 40min,STD	DAKO
ERG	EPR3864	X100,32min, Mild	abCAM
PTEN	Y184	X100, 16min, Mild	Gene Tex

*, pH 8.0, Mild, CC1 (pH 8.0 EDTA buffer) for 30 min; STD, CC1 (pH 8.0 EDTA buffer) for 60 min; Enzyme, protease; BMXT condition, Antigen retrieval condition and primary antibody incubation time; R.T.U., ready to us

Reactivity was detected by using an Ultra-View detection kit (Ventana Medical Systems, Tucson, AZ, USA). IHC for prostate secretory cells in the neoplastic and non-neoplastic glands was assessed as described previously [10]. The percentage of stained cells and the staining intensity in the nucleus and cytoplasm of malignant cells and paired benign cells were determined. Reactivity was graded from 0 to 4 according to these parameters. For all samples, IHC was performed and its results reviewed twice by two independent uropathologists, each with more than 15 years’ experience (WSP and GKL).

### Statistical methods

The immunostaining results for all 13 tumor markers were analyzed semi-qualitatively by three statisticians (BRP, and JJ). The Cox proportional hazards model with backward variable selection and an elimination criterion of 0.05 was used to identify significant predictors of BCR. Thirteen clinicopathological variables were assessed: age (years), resection margin, apical involvement, lymphovascular invasion, perineural invasion, seminal vesicle invasion, high grade intraepithelial neoplasm, pathologic T stage, tumor volume (mL), PSA level (ng/dL), prostate size (mL), Gleason score of the preoperative biopsy, and postoperative Gleason score. Age, prostate size, tumor volume, initial PSA level, time to BCR (months), and follow-up duration (months) were considered as continuous variables in the Cox regression analyses, whereas the other variables were considered as categorical variables.

After adjusting for the clinicopathologic variables that were significantly associated with BCR, the prognostic value of the 13 tissue biomarkers was evaluated by applying a backward variable selection method with an elimination criterion of 0.05 to a multivariable Cox proportional hazards model. Results were considered statistically significant when the p-values were two-sided and <0.05. Statistical analyses were performed by using SAS 9.3 software (SAS Institute Inc., Cary, NC, USA) and R software version 3.1.2.

## Results

During a median follow-up period of 57.5 months (range, 34–72 months), 67 (50.0%) patients experienced BCR (Table 2). The median time to BCR was 23 (13–53) months, and the median initial PSA level was 30.2 (16.3–56.6) ng/dL. The negative resection margin and no tumor (ypT0) rates were 75.4% (N = 101) and 3.0% (N = 4), respectively. Pathologic Gleason scores could not be determined in 93 (69.4%) patients owing to hormonal effects. Eight of the 41 (19.5%) patients with scores had upgraded scores, and eight (19.5%) had downgraded scores (data not shown). Other clinicopathological characteristics and the IHC staining results for the 13 biomarkers are summarized in Table 2.

**Table 2:** Summary of clinicopathological characteristics and immunohistochemical staining findings (N = 134).

Parameters	median (IQR) or N (%)
Age (years)	67 (62–70)
Resection margin positive	33 (24.6)
Apex involvement	11 (8.2)
Lymphovascular invasion	14 (10.5)
Perineural invasion	76 (56.7)
Seminal vesicle invasion	43 (32.1)
High grade intraepithelial neoplasm	7 (5.2)
Prostate size (mL)	25 (7–55)
Tumor volume (gm)	15 (10–30)
Initial PSA (ng/dL)	30.2 (16.3–56.6)
Neoadjuvant hormonal therapy	
LHRH agonist	17 (12.7)
LHRH + nonsteroidal antiandrogen	117 (87.3)
Biopsied Gleason Score	
6	31 (23.1)
7	59 (44.0)
8–10	44 (32.8)
Clinical stage	
cT1N0M0	7 (5.2)
cT2N0M0	75 (56.0)
cT3N0M0	40 (29.9)
cT4NxM0	12 (89.6)
Pathologic stage	
ypT0	4 (3.0)
ypT2	64 (47.8)
ypT3	47 (35.0)
ypT4 or N+	19 (14.2)
Postoperative Gleason Score	
Not available	93 (69.4)
6	11 (8.2)
7	16 (11.9)
8–10	14 (10.5)
Biochemical recurrence	67 (50.0)
Time to biochemical recurrence (months)	23 (13–54)
Follow-up duration (months)	57.5 (34–72)
RB loss	37 (27.6)
PTEN loss	52 (38.8)
ERG positive	7 (5.2)
C_erb2 positive	31 (23.1)
Cox2 positive	101 (75.9)
cyclinD1 positive	71 (53.0)
BCL2 positive	24 (17.9)
VEGF positive	125 (93.3)
PSCA positive	108 (80.6)
PMS positive	85 (63.4)
p53 positive	11 (8.2)
Ki67 positive	12 (9.0)
EGFR positive	21 (15.7)

The clinicopathological variables that predicted BCR, as determined by using the Cox proportional hazards model with backward variable selection, were as follows: seminal vesicle invasion [hazard ratio (HR), 2.39; 95% confidence interval (CI), 1.32–4.34; p = 0.004) [14], initial PSA level (HR, 1.01; 95% CI, 1.001–1.020; p = 0.002), prostate size (HR, 0.93; 95% CI, 0.90–0.97; p <0.001), and the Gleason score in the preoperative biopsy (HR, 1.34; 95% CI, 1.01–1.79; p = 0.046). After adjusting for these variables, a multivariable Cox proportional hazard analysis identified PSCA as a significant prognostic tissue biomarker for BCR (HR, 2.58; 95% CI, 1.06–6.27; p = 0.037) (Table 3). In a sub-analysis of the 41 samples with a Gleason score, two additional tissue markers significantly predicted BCR after adjusting for the clinicopathological variables listed above: PTEN loss (HR, 4.25; 95% CI, 1.23–14.74; p = 0.023) and Bcl-2 (HR, 10.86; 95% CI, 1.92–61.58; p = 0.007) (data not shown).

**Table 3:** Association of clinicopathological parameters and immunohistochemical staining with biochemical recurrence-free survival based on Cox Proportional Hazards Regression Models (N = 134).

	Univariable (N = 134, event = 67)			Multivariable(N = 134, event = 67)	
	HR (95% CI)		P-value	HR (95% CI)	P-value
Age		0.95 (0.93–0.98)	0.001		
Resection margin		2.20 (1.33–3.64)	0.002		
Apex involvement		2.09 (1.00–4.38)	0.047		
LVI		1.28 (0.58–2.80)	0.542		
PNI		1.37 (0.84–2.24)	0.210		
SVI		2.28 (1.38–3.75)	<.001	2.39 (1.32–4.34)	0.004
HGPIN		0.22 (0.03–1.57)	0.097		
Tumor volume		1.02 (1.01–1.02)	<.001		
PSA		1.01 (1.00–1.01)	<.001	1.01 (1.00–1.02)	0.002
Prostate size		0.94 (0.90–0.97)	<.001	0.93 (0.90–0.97)	<.001
pT3-4 Stage		2.41 (1.46–3.98)	<.001		
Biopsied GS		1.46 (1.18–1.82)	0.001	1.34 (1.01–1.79)	0.046
Postoperative GS		1.54 (1.02–2.32)	0.036		
RB loss		0.75 (0.41–1.38)	0.355		
PTEN loss		1.54 (0.95–2.50)	0.080		
ERG		1.16 (0.42–3.19)	0.774		
c_erb2		0.96 (0.55–1.69)	0.893		
cox2		0.97 (0.55–1.74)	0.928		
cyclinD1		0.89 (0.55–1.44)	0.641		
BCL2		1.51 (0.84–2.73)	0.166		
VEGF		0.74 (0.3–1.85)	0.519		
PSCA		1.11 (0.59–2.07)	0.750	2.58 (1.06–6.27)	0.037
PMS		1.00 (0.61–1.65)	0.994		
p53		1.73 (0.79–3.79)	0.165		
Ki67		1.66 (0.76–3.64)	0.200		
EGFR		1.08 (0.55–2.11)	0.828		

LVI, lymphovascular invasion; SVI, seminal vesicle invasion; PNI, perineural invasion; HGPIN, high-grade prostatic intraepithelial neoplasia; PSA, prostate specific antigen; pStage, pathologic stage; GS, Gleason score;

## Discussion

Although the benefits of NHT in patients with advanced PC include local control of the primary disease site with pathological complete remission and systemic control of microscopic metastases, they do not translate to better disease-free survival rates (assessed as lack of BCR or clinical progression) or overall survival rates [15]. This discrepancy is likely multi-factorial in nature and has galvanized the search for markers of biological responses with a clear impact on tumor morphology and immunohistochemical staining patterns [10–12, 15].

In this study, the expression of 13 PC-related proteins in RP specimens from 134 patients receiving NHT was evaluated via TMA with immunohistochemistry, a powerful recently developed tool that rapidly assesses the clinical significance of expressed molecular markers in human tumors including PC. Of these proteins, PSCA (HR, 2.58) was a significant indicator of early BCR in a multivariable analysis. In agreement with other studies [14, 16–20], we also identified several predictive clinicopathological variables: seminal vesicle invasion (HR, 2.39), initial PSA level (HR, 1.01), prostate size (HR, 0.93), and Gleason score in the preoperative biopsy (HR, 1.34) (all p-values <0.05, Table 3).

**Fig 1:**
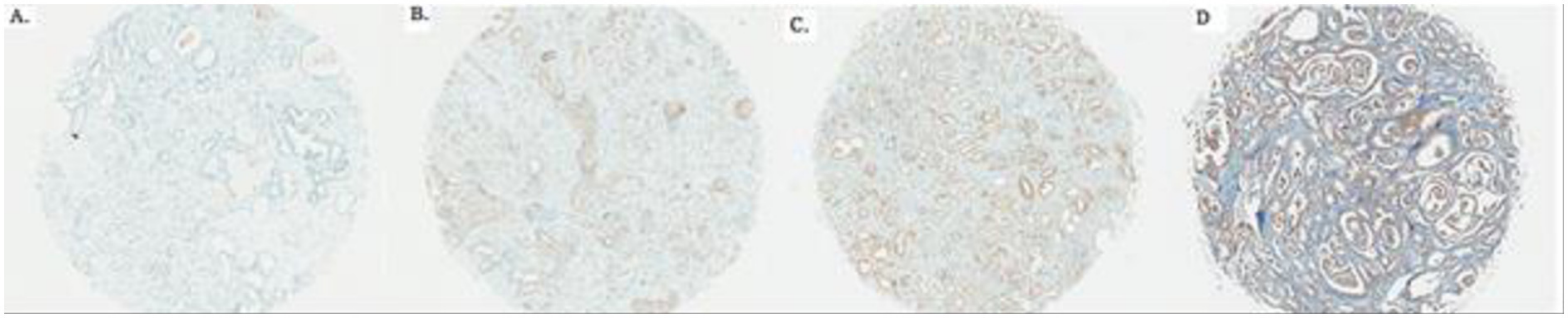
Representative immunohistochemical staining of prostate stem cell antigen (PSCA) in prostatectomy specimens of prostate cancers. Immunohistochemistry shows strong PSCA staining in the neoplastic epithelium from grades 0 to 3.

PSCA is a cell surface protein whose expression is very low in both normal and neoplastic prostate epithelium including metastatic bone lesions [21, 22]. It inhibits apoptosis and/or promotes proliferation during tumorigenesis and during the regrowth of androgen-independent PCs following their androgen-induced regression (i.e., during BCR) [21–23]. PSCA expression has shown been to correlate with the disease characteristics of PC (e.g., advanced tumor stage and high Gleason score) and with post-treatment clinical outcomes (e.g., progression to androgen-independent disease, local invasion, BCR, and bone metastases) [7, 24, 25]. Similar to previous studies, we showed that PSCA expression predicted a high risk of local recurrence after prostatectomy with NHT for PC; the BCR-free survival curves differed between the PSCA-positive and -negative groups after adjusting for the clinicopathological variables significantly associated with BCR (Table 3 and Fig 1).

Previous studies using either IHC, in situ hybridization, or circulating tumor cell assays [7, 23, 26] showed a good association between PSCA levels and clinical outcomes in PC. PSCA has been proposed as an alternative marker to PSA, because of its potential usefulness in predicting tumor biology, clinical prognosis, and especially treatment outcome after surgery, radiotherapy, or hormonal therapy [27, 28]. Despite its sensitivity and popularity as a marker of the disease state, progression, and pre-treatment outcomes of PC [29], PSA has certain limitations. Its accuracy in predicting post-treatment outcomes is compromised by its fluctuating levels, and it does not clearly distinguish between benign disease and PC. These weaknesses of PSA as a prognostic indicator might be compensated by also considering PSCA levels. Evaluating the levels of both markers might increase the accuracy of predicting the clinical outcome of PC.

NHT usually decreases cell proliferation (as indicated by a decrease in the percentage of Ki-67-labeled cells) and diploidy, induces apoptosis and consequent atrophy of non-neoplastic and neoplastic prostatic epithelium as characterized by the fragmentation of tumor DNA and the appearance of apoptotic bodies, and downregulates the expression of vessel-related markers such as vascular endothelial growth factor [30, 31]. The mechanisms underlying these events are not fully understood, but may involve several oncoproteins, tumor suppressor proteins, and growth factors, such as Ki-67, p53, Bcl-2, and PTEN [32].

In this study, other tissue markers except for PSCA were not significant prognostic tissue markers of BCR-free survival (p >0.05, Table 3). However, when the 41 samples with Gleason scores were analyzed separately, PTEN loss (HR, 4.25) and Bcl-2 (HR, 10.86) were significant predictive after adjusting for the significant clinicopathological variables (p<0.05, data not shown). Both events have been previously associated with disease progression and androgen dependence in PCs after androgen withdrawal therapy [31, 32]. PTEN inhibits the transcriptional activity of the androgen receptor in androgen-sensitive PC cells and increases the anti-proliferative effects of anti-androgens, perhaps via an androgen receptor-independent mechanism, in androgen-independent PC cells. Bcl-2 is frequently overexpressed in PCs, and its association with both hormonal therapy and chemotherapy resistance is well known [33].

Many pathologists discourage Gleason scoring after androgen ablation because androgen ablation creates histological alterations that may falsely indicate high scores [34]. Gleeve et al. [35] reported that Gleason scores increase from 8 to 10 in 10% of patients receiving NHT plus RP; in our study, eight of 41 (19.5%) patients had upgraded scores, and 8 (19.5%) had downgraded scores.

NHT can decrease the rate of positive surgical margins, but does not influence the rate of BCR or the survival rates in patients. Patients must be rigorously examined after NHT for residual carcinoma to determine whether the disease has progressed or regressed, to assess the status of the surgical margin, and to select the appropriate follow-up modalities, because histological assessment of androgen-ablated prostates is inaccurate. Margins considered negative according to HE staining after NHT plus RP may be deemed positive (as indicated by the presence of residual tumor cells) according to cytokeratin staining in androgen-ablated prostates, especially in the peripheral zone, small glands, and fibrous stroma [31]. In this study, the absence of residual carcinoma was confirmed in 4 (3.0%) of the 134 specimens via both HE and cytokeratin staining, and resection margin was not a significant prognostic indicator of BCR in the multivariable analysis (p >0.05, Table 3). However, the present study has some limitations. It was retrospective, and the follow-up period was not long enough for the assessment of survival rates. Processing artifacts in IHC, such as the shrinkage of prostate specimens, may have occurred.

## Conclusion

NHT is an optional therapy for a subset of patients with high risk PC. The tissue biomarker PSCA, as examined in prostatectomy specimens, was a significant prognostic indicator of early BCR, as were some clinicopathological factors, in patients with high risk PC receiving NHT.
